# Synthesis of Silver Nanoparticles in Chitosan, Gelatin and Chitosan/Gelatin Bionanocomposites by a Chemical Reducing Agent and Their Characterization

**DOI:** 10.3390/molecules16097237

**Published:** 2011-08-25

**Authors:** Mansor Bin Ahmad, Jenn Jye Lim, Kamyar Shameli, Nor Azowa Ibrahim, Mei Yen Tay

**Affiliations:** Department of Chemistry, Faculty of Science, Universiti Putra Malaysia, UPM Serdang, Selangor 43400, Malaysia; E-Mails: jennjye87@yahoo.com (J.J.L.); kamyarshameli@gmail.com (K.S.); norazowa@science.upm.edu.my (N.A.I.); tmyen_87@hotmail.com (M.Y.T.)

**Keywords:** chitosan, gelatin, silver, bionanocomposite, chemical reduction method

## Abstract

In this research, silver nanoparticles (AgNPs) were synthesized in chitosan (Cts), Cts/gelatin and gelatin suspensions using a chemical reducing agent. Cts and gelatin were used as natural stabilizers and solid support, whereas AgNO_3_ was used as the silver precursor. Sodium borohydride (NaBH_4_) was used as the reducing agent. The properties of AgNPs in Cts, Cts/gelatin and gelatin bionanocomposites (BNCs) were studied in terms of their surface plasmon resonance, crystalline structure, average diameter size, particle distributions, surface topography and functional groups. All the samples were characterized by UV-visible spectroscopy, powder X-ray diffraction, transmission electron microscopy, atomic force microscopy and Fourier transform infrared spectroscopy.

## 1. Introduction

The field of nanotechnology has emerged as one of the most active areas of research in materials science over the last decades. The fact that nanoparticles (NPs) exhibit quite a number of interesting and unique properties based on their specific characteristics, such as size, distribution and morphology leads itself to various applications [1], for instance in catalysis, microelectronics, sensors [2], biomaterials, therapeutics [3] as well as antimicrobial [4] applications. The term nanocomposite was derived to define filled polymers containing dispersed nanoparticles. Based on the definition, another term has been derived which is bionanocomposites (BNCs). BNCs are made of a natural polymeric matrix and inorganic/organic filler with at least one dimension on the nanometer scale. Metal/polymer compounds such as BNCs are becoming a promising new research field for their unique properties [5].

During the preparation of silver nanoparticles (AgNPs), stabilizers are one kind of agent that should be present and that play an important role in controlling the formation of nanoparticles, as well as their dispersion stability. Polymers are often used as particle stabilizers due to the fact that they are effective in preventing agglomeration and precipitation of the particles. This is important in synthesizing nanoparticles with homogenous distributions. Various methods have been introduced to synthesize Ag nanocomposites, including photochemical [5,6,7], chemical [3,8], microwave [9], sono-chemical [10,11], and radiochemical reducing methods [12,13]. Synthesis of AgNPs using different methods has been carried out with increasing frequency. This further supports the fact this field is currently receiving a lot of attention. Some examples of composites being synthesized include AgNPs with polymers such as poly(vinylalcohol) [14], poly(vinylpyrolidone) [13,15], polymethylmethacrylate [16], poly(lactic acid) [17], poly(methyl vinyl ether) [18] chitosan [19,20], gelatin [21] *etc*. This accounts for the different properties of composites that are synthesized, which allow them to serve different purposes in our daily life.

Chitosan (Cts) is a polysaccharide that occurs naturally. Its units are composed of randomly distributed β-(1-4)-linked D-glucosamine (deacetylated unit) and *N*-acetyl-D-glucosamine (acetylated unit). Cts has been investigated as a natural cationic biopolymer because of its known excellent biocompatibility, biodegradability, nontoxicity, bioactivity, and multifunctional groups from years of research. It is also extensively being studied for food packaging film, bone substitutes, artificial skin, biomedical applications and pH sensitive drug delivery among others due to a number of great properties it possesses [22].

Gelatin is made up of a mixture of peptides and proteins produced by partial hydrolysis of naturally occurring collagen. The carboxyl groups on its chain backbones are one of its amazing features that enables the possibility to form hydrogen bonds with chitosan for a well-mixed hybrid [23]. Besides, the fact that it is used in pharmaceuticals, cosmetic manufacturing among others prove that it is perfectly non-toxic to human. This explains the preferable usage of gelatin in research work as potential side effects of compounds synthesized from it as precursor can be reduced to a minimum. 

Here, we report synthesis of AgNPs by chemical reduction method using NaBH_4_. Cts, Cts/gelatin and gelatin suspension were used with the original intention to prevent aggregation of AgNPs. In addition, these matrixes were found to be functioning as stabilizer also in later stage of experiment. The size of the silver nanoparticles synthesized is less than 15 nm.

## 2. Results and Discussion

In this research, Cts, Cts/gelatin and gelatin suspension were used as the stabilizers for reducing AgNO_3_ using NaBH_4_ as strong reducing agent. As a result, AgNO_3_ was successfully reduced by NaBH_4_ in the presence of Cts/gelatin or either one, resulting in the formation of AgNPs according to the following equations (1–3) [24]:

Ag^+^/Cts + BH_4_^−^ + 3H_2_O → Ag^0^/Cts + B(OH)_3_ + 3.5H_2_ (1)

Ag^+^/Cts/gelatin + BH_4_^−^ + 3H_2_O → Ag^0^/Cts/gelatin + B(OH)_3_ + 3.5H_2_ (2)

Ag^+^/gelatin + BH_4_^−^ + 3H_2_O → Ag^0^/gelatin + B(OH)_3_ + 3.5H_2_ (3)

**Figure 1:**
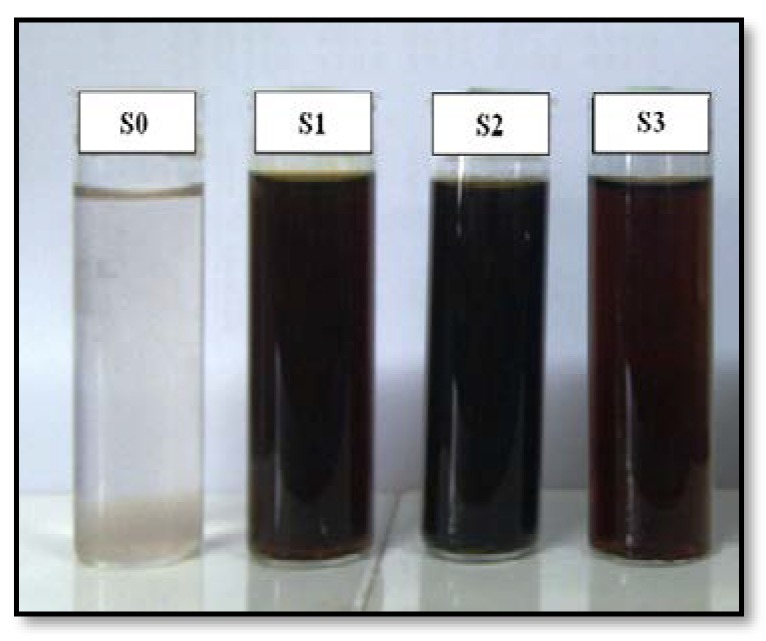
Photograph of AgNO_3_/Cts/gelatin (S0), Ag/Cts (S1), Ag/Cts/gelatin (S2) and Ag/gelatin (S3) bionanocomposite suspensions.

**Figure 2:**
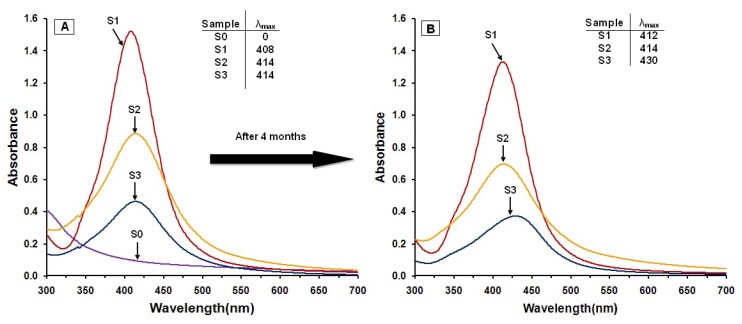
Ultraviolet-visible absorption spectra of AgNO_3_/Cts/gelatin (S0), Ag/Cts (S1), Ag/Cts/gelatin (S2), and Ag/gelatin (S3) BNCs suspension after few hours (**A**); and after four months (**B**).

**Figure 3:**
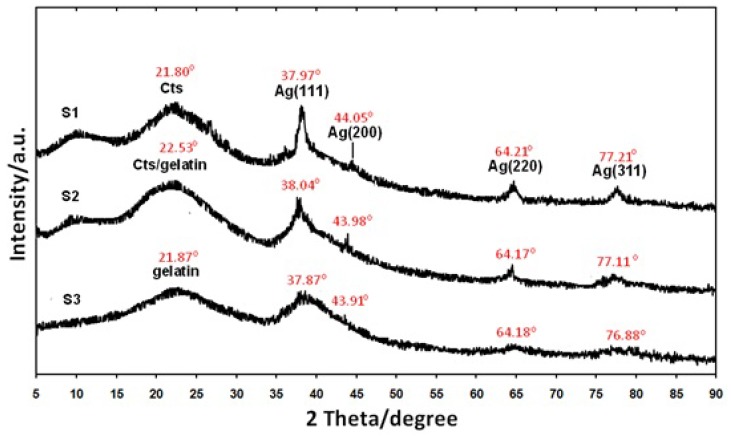
Powder X-ray diffraction patterns of Ag/Cts (S1), Ag/Cts/gelatin (S2) and Ag/gelatin (S3) BNCs for its crystals structure determination.

As shown in Figure 1, the AgNO_3_/Cts/gelatin suspensions (S0) were colorless. After the addition of the reducing agent to the suspensions, they turned dark brown (S1-S3) indicating the formation of AgNPs in the Cts, Cts/gelatin and gelatin suspensions. The formation of AgNPs was also followed by measuring the surface plasmon resonance bands of the AgNO_3_/Cts/gelatin (S0), Ag/Cts (S1), Ag/Cts/gelatin (S2) and Ag/gelatin (S3) suspensions at wavelengths within the range of 300–700 nm (Figure 2). The PXRD patterns in the wide angle range of 2θ (5° < 2θ < 90°) were also detected for the crystalline structures determination of the synthesized Ag NPs (Figure 3).

**Figure 4:**
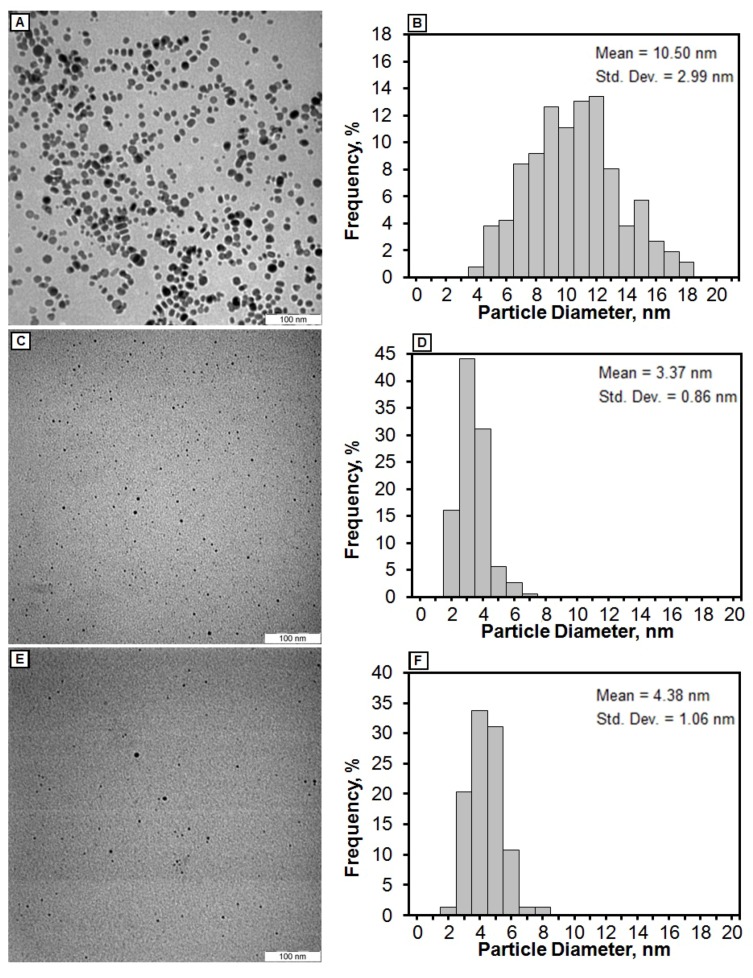
Transmission electron microscopy images and corresponding particle size distributions of Ag/Cts (**A**,**B**), Ag/Cts/gelatin (**C**,**D**) and Ag/gelatin (**E**,**F**) BNCs.

**Figure 5:**
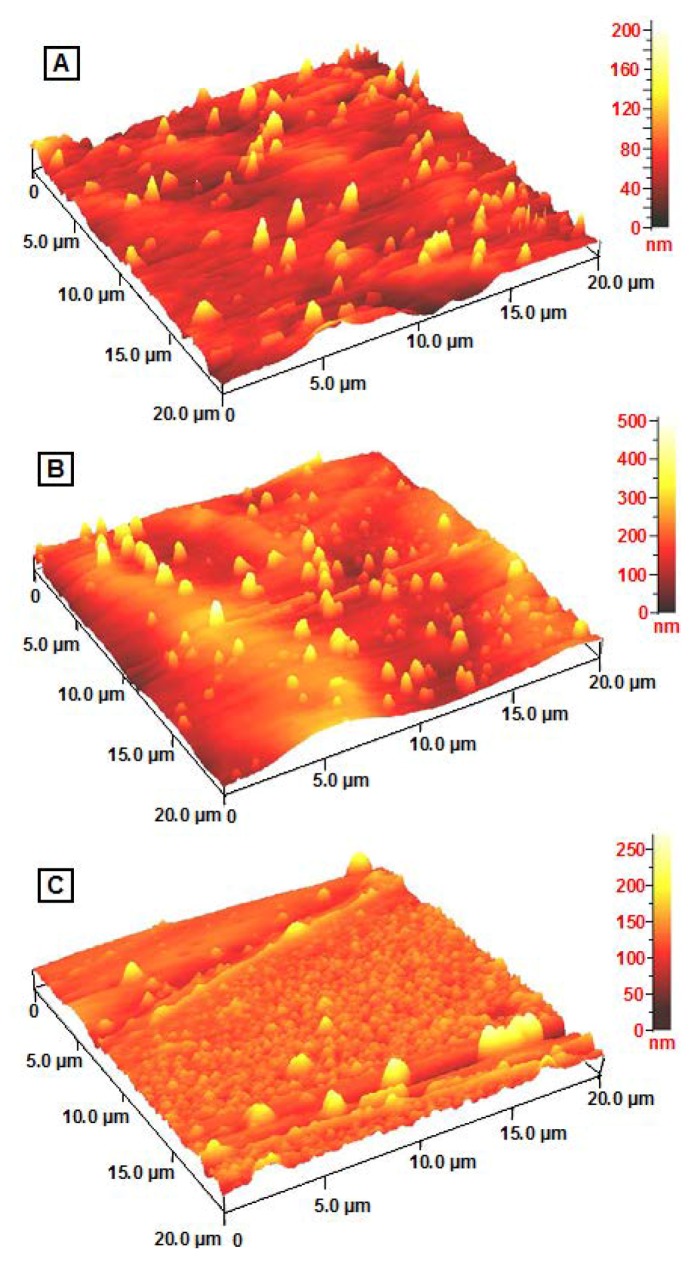
AFM images of the surface topography of Ag/Cts (**A**); Ag/Cts/gelatin (**B**); and Ag/gelatin (**C**) thin films.

**Figure 6:**
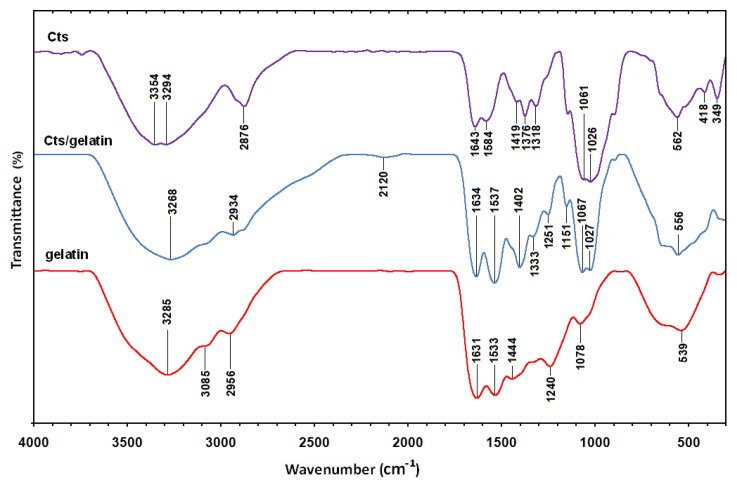
Fourier transform infrared spectra of Cts, Cts/gelatin, and gelatin.

**Figure 7:**
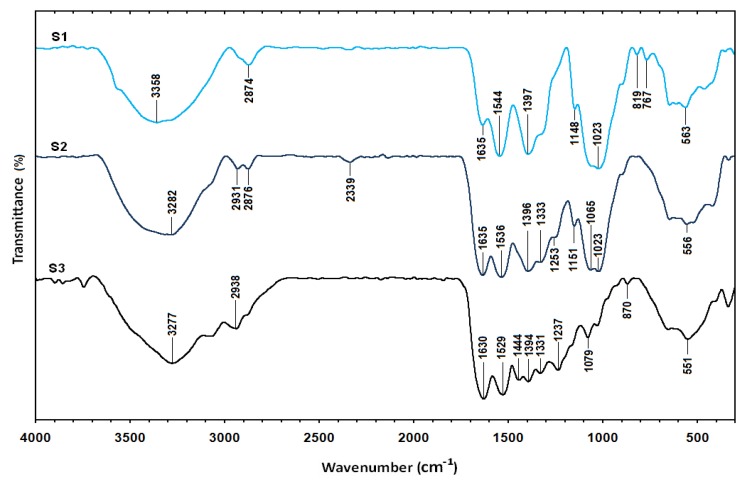
Fourier transform infrared spectra for the Ag/Cts (S1), Ag/Cts/gelatin (S2) and Ag/gelatin (S3).

The TEM images and size distribution of the AgNPs showed that the mean diameter of the nanoparticles ranged from about 3.37 nm to 10.50 nm (Figure 4). As shown in Figure 5, the AFM images show additional information for the nanoparticles size detection. The chemical structures of pure polymers and AgBNCs were analyzed by FTIR spectroscopy (Figure 6 and Figure 7).

### 2.1. Ultraviolet-Visible Spectroscopy

The color of the AgNO_3_/Cts/gelatin suspensions which undergo the NaBH_4_ reduction process changed from colorless to dark brown. These color changes indicated that the AgNPs were formed in the Cts, Cts/gelatin and gelatin suspension. This statement is further proven by UV-visible spectrum in Figure 2A. No absorption band is observed in the 300–500 nm range for AgNO_3_/Cts/gelatin, showing that no AgNPs formation has occurred in the sample. After the addition of NaBH_4_, surface plasmon resonance bands were detected around 408–414 nm, indicating the formation of AgNPs. These absorption bands were an indication that the diameters of the AgNPs were small [25]. These results were further confirmed with the TEM data, revealing the diameter of the AgNPs. The absorption peak of Ag for Ag/Cts BNC suspension with value of 408 nm was red-shifted to a higher wavelength (414 nm) in Ag/Cts/gelatin and Ag/gelatin sample. The SPR absorption band and TEM results showed signs of an exceptional case: as size decreases from d = 10.50 nm it blue-shifts, but then turns over near d = 3.37 and 4.38 nm and it red-shifts. It could be explained by the Mie theory model which indicates that the reason of the red-shift is the lowered conductivity in the outer metallic layer caused by chemical interactions [26]. For the stability test of the AgNPs, absorption spectra of the samples were measured after storage for four months (Figure 2B). The absorption peaks of the Ag NPs in S1 and S3 shift slightly from 408 to 412 nm and 414 to 430 nm respectively, whereas the absorption peak for the S2 remains the same at 414 nm. Overall, the spectra for all the samples showed no significant changes in either their intensity or spectral shape [27].

### 2.2. Powder X-Ray Diffraction

As shown in Figure 3, all the AgBNCs had a similar x-ray diffraction patterns. The PXRD peaks at 2θ with value of around 38°, 44°, 64°, and 77° could be recognized to the 111, 200, 220, and 311 crystallographic planes of the face-centered cubic Ag crystals, respectively [28]. For Ag/Cts BNCs, its PXRD profile showed peak at 37.97°, 44.05°, 64.21° and 77.21° related to AgNPs whereas peak at 21.80° related to Cts. PXRD profile of Ag/Cts/gelatin BNCs displayed peak at 22.53° for Cts/gelatin, 28.04°, 43.98°, 64.17° and 77.11° for Ag. Lastly, the Ag/gelatin BNCs showed peak at 21.87° for gelatin, 37.87°, 43.91°, 64.18° and 76.88° for Ag crystals structure. These results further confirmed the existence of AgNPs as final product in the Cts, Cts/gelatin and gelatin BNCs 

### 2.3. Morphology

The TEM images of AgBNCs and the particle size distributions for Ag/Cts, Ag/Cts/gelatin and Ag/gelatin are shown in Figure 4. TEM images for those three samples revealed the mean diameters with standard deviation of the AgNPs about 10.50 ± 2.99, 3.37 ± 0.86, and 4.38 ± 1.06 nm for Ag/Cts (Figures 4A,B), Ag/Cts/gelatin (Figures 4C,D) and Ag/gelatin (Figures 4E,F), respectively. These results showed that the diameters of the AgNPs are the smallest in Cts/Gelatin suspension compared to Cts and Gelatin alone. In addition, the TEM image of Ag/Cts showed some aggregation of AgNPs, whereas the TEM image of Ag/gelatin showed fewer AgNPs within the gelatin suspension. With the combination of both Cts and gelatin, the TEM image of Ag/Cts/gelatin illustrated the good distribution and higher occurrence of the AgNPs. 

Thin films of Ag/Cts and Ag/gelatin present different surface topography when analysed with AFM (Figures 5A and 5C). However the Ag/Cts/gelatin film exhibits combination of both Cts and gelatin surface topography (Figure 5B). The AgBNCs film morphologies were dependent on several factors including polymer solubility, solvent evaporation, total thickness, molecular weight and surface composition [29].

### 2.4. Fourier Transform Infrared Spectroscopy

The Fourier transform infrared spectrum of Cts (Figure 6) shows vibration bands at 3354 and 3294 cm^−1^ due to overlapping of O–H and amine N–H stretching bands; a peak at 2876 cm^−1^ indicated aliphatic C–H stretching; 1643 and 1584 cm^−1^ for N–H bending; 1419, 1376, and 1318 cm^−1^ for C–H bending; and 1061 and 1026 cm^−1^ for C–O stretching [30]. The spectrum of gelatin (Figure 6) showed vibration bands at 3285 cm^−1^ for N–H stretch coupled with hydrogen bonding (HB), 3085 cm^−1^ for alkenyl C–H stretch, 2956 cm^−1^ for CH_2_ asymmetrical stretching, 1631 cm^−1^ for C=O stretch/HB coupled with COO^−^, band at 1533 cm^−1^ for N–H bend coupled with CN stretch, 1444 cm^−1^ for CH_2_ bend, 1240 cm^−1^ for NH bend, and 1078 cm^−1^ for C–O stretch [31]. The spectra of Cts/gelatin composite showed a mixture of characteristic absorptions due to the amine groups of Cts and the carboxylic acid groups of gelatin. The amine group peaks for pure chitosan were shifted from 1643 and 1584 cm^−1^ to 1634 and 1537 cm^−1^ in Cts/gelatin composite, corresponding to the bend vibration of amine chitosan. The pure gelatin was characterized by an amino band at 1533 cm^−^^1^ and a carbonyl peak at 1631 cm^−^^1^ shifted to 1634 and 1537 cm^−1^ as well. The shifting of both amino and carbonyl bands in the spectra of Cts/gelatin composite showed the formation of complex involving HB in between chitosan and gelatin [23]. The spectrum of Ag/Cts BNCs (Figure 7) showed a blue shift of the Cts peaks at 1643 and 1584 cm^−^^1^ to 1635 and 1544 cm^−^^1^, respectively, and also displayed a high intensity peak at 1544 cm^−^^1^. The vibration band of Ag/Cts at 1397 cm^−^^1^ could indicate the interaction between AgNPs with Cts. The spectrum of Ag/Cts/gelatin BNCs (Figure 7) presented a narrower vibration band for the O–H group at 3282 cm^−^^1^ compared to Cts/gelatin composite and also the interaction peak between Ag and Cts/gelatin at 1396 cm^−^^1^. In the spectrum of Ag/gelatin BNCs, the occurrence of the vibration band at 1394 cm^−^^1^ it is also noted.

## 3. Experimental

### 3.1. Materials

All reagents in this work were of analytical grade and were used as received without further purification. AgNO_3_ (99.98%) was used as the silver precursor, and was obtained from Merck (Darmstadt, Germany). Meanwhile, NaBH_4_, the reducing agent, was obtained from Sigma–Aldrich (St. Louis, MO, USA). Low molecular weight chitosan and glacial acetic acid (99%) were also obtained from Sigma–Aldrich. The gelatin was obtained from HiMedia (Bombay, India). All the aqueous solutions were prepared with double-distilled water. 

### 3.2. Synthesis AgNPs in Chitosan BNC

The chitosan suspension was prepared by solubilizing chitosan (1.0 g) in acetic acid (50 mL, 1.0 wt %) solution. Then, AgNO_3_ (50 mL, 0.01 M) was added immediately into the suspension under constant stirring for 2.0 hours for preparation of the AgNO_3_ in chitosan suspension. NaBH_4_ (20 mL, 0.04 M) was added to the suspension of AgNO_3_/Cts and an immediate color change from pale yellow to brown indicating the formation of AgNPs was noted. This suspension was stirred for 1.0 hour more, then the obtained Ag/Cts BNCs were finally made into thin films for further characterization. 

### 3.3. Synthesis AgNPs in Chitosan/Gelatin BNC

Chitosan (0.5 g) was dissolved in acetic acid (25 mL, 1.0 wt %) solution. On the other hand, gelatin (0.5 g) was dissolved in warm (40 °C) distilled water (25 mL). Next, both suspensions were mixed together and AgNO_3_ (50 mL, 0.01 M) was added directly into the suspensions. The AgNO_3_/Cts/gelatin suspension was stirred for 2.0 hours. The NaBH_4_ (20 mL, 0.04 M) was added immediately to the suspension of AgNO_3_/Cts/gelatin. A color change from colorless to dark brown was observed. The suspension was stirred for another 1.0 hour, then the obtained Ag/Cts/gelatin BNCs were made into thin films for characterization. 

### 3.4. Synthesis AgNPs in Gelatin BNC

For preparation of gelatin suspension, gelatin (1.0 g) was solubilized in warm (40 °C) distilled water (50 mL). After that, AgNO_3_ (50 mL, 0.01 M) was mixed into the suspension and stirring continued for 2.0 hours. NaBH_4_ (20 mL, 0.04 M) was introduced immediately to the suspension of AgNO_3_/gelatin. An immediate color change from colorless to light brown was observed. This suspension was stirred for 1.0 hour. The product was finally prepared in thin films for further characterization. 

### 3.5. Characterization Methods and Instruments

The prepared Ag BNCs were characterized by UV-visible spectroscopy (UV-visible), powder X-ray diffraction (PXRD), transmission electron microscopy (TEM), atomic force microscopy (AFM), and Fourier transform infrared spectroscopy (FTIR). The UV-visible spectra were detected over the range of 300–700 nm using a Shimadzu H.UV.1650 PC UV-visible spectrophotometer. Crystalline structures of the synthesized Ag were examined using Philips X’pert Pro Panalytical PW3040MPD X-ray diffraction. TEM image observations were carried out on Hitachi H–7100 electron microscope and the particle size distributions were determined using the UTHSCSA Image Tool program (V. 3.00; University of Texas Health Science Center, San Antonio, TX, USA). FTIR spectra were recorded over the range of 300–4000 cm^−1^ using a Series 100 Perkin Elmer FT-IR 1650 spectrophotometer. The AFM image was obtained on an Ambios Q-scope (Ambios Technology, Santa Cruz, CA, USA) (SPM) machine.

## 4. Conclusions

AgNPs were successfully introduced into Cts, Cts/gelatin, and gelatin natural polymers using a chemical reduction method. This was confirmed by the maximum surface plasmon resonance peak at 408–414 nm for each sample indicating the formation of Ag NPs. The crystalline structures of AgNPs were found to be face centered cubic type. The average diameters of the AgNPs were revealed to be 10.50, 3.37 and 4.38 nm for Ag/Cts, Ag/Cts/gelatin and Ag/gelatin, respectively. The best distribution of AgNPs was found to be in Cts/gelatin suspension and their average diameters were found to be the smallest from TEM results. Hence, we conclude that Cts/gelatin works better as stabilizer and size control compared to Cts and gelatin alone.
